# AI- vs Human-Based Assessment of Medical Interview Transcripts in a Generative AI–Simulated Patient System: Cross-Sectional Validation Study

**DOI:** 10.2196/81673

**Published:** 2026-02-17

**Authors:** Hiromizu Takahashi, Kiyoshi Shikino, Takeshi Kondo, Yuji Yamada, Yoshitaka Tomoda, Minoru Kishi, Yuki Aiyama, Sho Nagai, Akiko Enomoto, Yoshinori Tokushima, Takahiro Shinohara, Fumiaki Sano, Takeshi Matsuura, Rikiya Watanabe, Toshio Naito

**Affiliations:** 1 Department of General Medicine, Faculty of Medicine, Juntendo University, 3-1-3 Hongo, Bunkyo Tokyo, 1130033 Japan, Tokyo, Japan, 81 3-3813-3111; 2 Department of Community-Oriented Medical Education, Graduate School of Medicine, Chiba University, Chiba, Japan; 3 Center for Postgraduate Clinical Training and Career Development, Nagoya University Hospital, Nagoya, Japan; 4 The School of Health Professions Education, Maastricht University, Maastricht, The Netherlands; 5 Brookdale Department of Geriatrics and Palliative Medicine, Icahn School of Medicine at Mount Sinai, New York, NY, United States; 6 Department of General Internal Medicine, Itabashi Chuo Medical CenterTokyo, Japan; 7 Department of Internal Medicine, Nishiwaki Municipal Hospital, Hyogo, Japan; 8 Anesthesiology and Critical Care Medicine, Tenri Hospital, Nara, Japan; 9 Department of Nursing, School of Nursing, University of Human Environments, Aichi, Japan; 10 Department of General Medicine, Saga University Hospital, Saga, Japan; 11 Department of General Medicine, Graduate School of Medical and Dental Sciences, Institute of Science Tokyo, Tokyo, Japan; 12 Department of General Medicine, Bibai City Hospital, Hokkaido, Japan; 13 Department of General Internal Medicine, Kita-Harima Medical CenterHyogo, Japan

**Keywords:** medical education, artificial intelligence, AI, virtual patient, clinical interview, ChatGPT, simulation-based learning

## Abstract

**Background:**

Generative artificial intelligence (AI) is increasingly used in medical education, including AI-based virtual patients to improve interview skills. However, how much AI-based assessment (ABA) differs from human-based assessment (HBA) remains unclear.

**Objective:**

This study aimed to compare the quality of clinical interview assessments generated via an ABA (GPT-o1 Pro [ABA-o1] and GPT-5 Pro [ABA-5]) with those generated via an HBA conducted by clinical instructors in an AI-based virtual patient setting. We also examined whether AI reduced evaluation time and assessed agreement across participants with different levels of clinical experience.

**Methods:**

A standardized case of leg weakness was implemented in an AI-based virtual patient. Seven participants (2 medical students, 3 residents, and 2 attending physicians) each conducted an interview with the AI patient, and transcripts were scored using the 25-item Master Interview Rating Scale (0‐125). Three evaluation strategies were compared. First, GPT-o1 Pro and GPT-5 Pro scored each transcript 5 times with different random seeds to test case specificity. Processing time was logged automatically. Second, 5 blinded clinical instructors independently rated each transcript once using the same rubric. Third, reliability metrics were applied. For AI, intraclass correlation coefficients (ICCs) quantified repeatability. For humans, the ICC(2,1) was calculated. Agreement was quantified using the Pearson *r*, Lin concordance correlation coefficient, Bland-Altman limits of agreement, Cronbach α, and ICC. Time efficiency was expressed as mean minutes per transcript and relative percentage reduction.

**Results:**

Mean interview scores were similar across methods (ABA-o1: mean 52.1, SD 6.9; ABA-5: mean 53.2, SD 6.8; HBA: mean 53.7, SD 6.8). Agreement between ABA and HBA was strong (*r*=0.90; concordance correlation coefficient=0.88) with minimal bias (ABA-o1: mean 0.4, SD 2.7; ABA-5: mean 1.5, SD 5.2; limits of agreement: –4.9 to 5.7 for ABA-o1 and –8.6 to 11.7 for ABA-5). The Cronbach α was 0.81 (ABA-o1), 0.86 (ABA-5), and 0.80 (HBA); the ICC(3,1) was 0.77 (ABA-o1) and 0.82 (ABA-5); and the ICC(2,1) was 0.38 (HBA). The coefficient of variation for ABA was approximately half that of HBA (6.6% vs 13.9%). Processing time for 5 runs was 4 minutes, 19 seconds for ABA-o1 and 3 minutes, 20 seconds for ABA-5 vs 10 minutes, 16 seconds for physicians, corresponding to 58% and 67.6% reductions, respectively.

**Conclusions:**

ABA-o1 and ABA-5 produced scores closely matching HBA while demonstrating superior consistency and reliability. In the setting of virtual interview transcripts, these findings suggest that ABA may serve as a valid, rapid, and scalable alternative to HBA, reducing per-assessment time by over half. Applied strategically, AI-based scoring could enable timely feedback, improve efficiency, and reduce faculty workload. Further research is needed to confirm generalizability across broader settings.

## Introduction

### Background

Effective clinical interviewing is essential for making correct diagnoses and building strong relationships with patients [1]. Traditionally, students learn these skills through supervised practice with real or standardized patients and feedback from faculty [1]. However, this apprenticeship-style approach is time-intensive and limits opportunities for deliberate practice [2].

The assessment component itself also consumes substantial faculty and resident physician (RP) time. In competency-based medical education (CBME), faculty complete numerous workplace-based assessment forms; one Canadian study found a mean of 3 minutes, 6 seconds per entrustable professional activity form, adding approximately 18 minutes of extra documentation time for each staff member every 4-week block [3]. Multiprogram qualitative work further confirms that the cumulative “assessment burden” is now viewed as a major threat to sustainability, prompting programs to redesign processes to reduce administrative load [4].

Recently, generative artificial intelligence (AI) using large language models (LLMs) has enabled the creation of AI-based virtual patients that both converse with learners and automatically evaluate performance [2,5]. Empirical studies have shown promising results for AI assessment in free-text clinical documentation [6], script concordance testing [7], and objective structured clinical examination (OSCE) history-taking stations [8]. Many of these systems use validated rubrics such as the Master Interview Rating Scale (MIRS) to structure feedback [9]. Nevertheless, the reliability and validity of AI-generated ratings remain understudied; therefore, establishing concordance with expert evaluations is a prerequisite for educational or licensure use.

### Objectives

This study compared AI-based assessment (ABA) scores of clinical interview performance using GPT-o1 Pro (OpenAI; ABA-o1) and GPT-5 Pro (OpenAI; ABA-5) with human-based assessment (HBA) scores. We hypothesized that ABA scores and HBA scores would exhibit strong concordance and that ABA scoring would serve as a substitute for HBA scoring. We also hypothesized that AI would complete evaluations more rapidly, reducing the assessment burden on clinicians. A secondary aim was to evaluate agreement across participants with differing clinical experience and evaluate whether the use of AI could lead to a measurable reduction in evaluation time, thereby contributing to overall efficiency in assessment processes.

## Methods

### Study Design and Setting

A cross-sectional validation study was conducted. This study involved 3 medical students (MSs), 3 RPs, and 2 attending physicians (APs) who participated in standardized clinical scenarios.

### Virtual Patient Scenario

A man aged 27 years presenting with progressive bilateral leg weakness, particularly proximal, was scripted based on a published case of thyrotoxic periodic paralysis. The scenario, created by a general internal medicine specialist with extensive educational experience drawing directly on prior literature, included relevant clinical history (eg, recent myalgias, tremors, diarrhea, and insomnia), red flag cues (eg, acute onset, muscle weakness, and hypokalemia), and psychosocial factors (eg, recent immigration and use of herbal supplements). The case represented a classic presentation of thyrotoxic periodic paralysis caused by hyperthyroidism. The patient was implemented as an AI-simulated character using ChatGPT’s custom generative pretrained transformers.

### Participants

The participants were recruited through convenience sampling complemented by snowball sampling. MSs comprised a third-year and a fifth-year student; RPs comprised 3 postgraduate year 1 residents; and APs comprised 2 board-certified physicians in internal medicine or general internal medicine in Japan, each with ≥5 years of clinical teaching experience.

Each participant conducted a history-taking encounter by speaking with an AI patient. All conversations were recorded and transcribed verbatim. As all interviews took place within the ChatGPT-based simulated patient interface, transcripts were automatically generated from dialogue logs without manual correction.

### Scoring Instrument

The MIRS from the University of Tennessee was originally designed to assess 27 items. In this study, 25 of these items were evaluated based on the available conversational recordings. Each item was rated on a scale from 0 to 5 (total possible score: 0‐125) covering domains such as information gathering, organization, empathy, and patient-centered communication. The excluded items were nonverbal behavior and pace and flow of the interview, which require audiovisual input to evaluate.

### Assessment Methods

The main outcome was the comparison of MIRS scores.

For ABA-o1, each transcript was submitted separately to GPT-o1 Pro with a base prompt directing it to rate the encounter using the MIRS and justify each score. This process was repeated 5 times per transcript, and item-level and total scores were averaged across runs. For ABA-5, using the same base prompt, the 7 transcripts were scored in 2 batch submissions rather than individually. Run 1 included MS 1, MS 2, RP 1, and RP 2, and run 2 included RP 3, AP 1, and AP 2. For each batch, the prompt explicitly stated that it contained 4 interview transcripts (run 1) or 3 interview transcripts (run 2). For each participant within a batch, item-level and total MIRS scores were extracted from the model’s output. The base prompt and model settings were held constant across runs, and the full prompt is provided in Multimedia Appendix 1 to support reproducibility. For both ABA-o1 and ABA-5, reproducibility was further examined by scoring each transcript 5 times using different random seeds.

For HBA, 5 blinded clinical instructors independently rated each transcript using the same MIRS rubric. All assessors were board certified in general internal medicine or general medicine in Japan, actively involved in medical education, and coauthors of this study (Y Tokushima, TS, RW, TM, and FS). Item-level and total scores were averaged across the 5 raters. To further ensure calibration beyond the preparatory webinar, raters briefly discussed scoring rationales for sample transcripts to reach consensus on the interpretation of rubric items.

The secondary outcome was the comparison of assessment time, which was assessed as follows:

- Physician scoring time for HBA—a stopwatch measured the time from transcript review to completion of scoring.
- AI scoring time for ABA-o1—the elapsed time was automatically recorded for each of the 7 individual submissions from prompt submission to receipt of the complete output.
- AI scoring time for ABA-5—the elapsed time was automatically recorded for each of the 2 batch submissions from prompt submission to receipt of the complete output.

For all 3 methods, mean assessment time and SD were calculated, and absolute and relative time savings of ABA vs HBA were reported.

### Statistical Analysis

All analyses used R (version 4.3.1; R Foundation for Statistical Computing). Descriptive statistics (mean and SD and coefficient of variation [CV]) summarized the scores. Agreement was assessed using the Pearson correlation coefficient (*r*) for linear associations; the Lin concordance correlation coefficient (CCC) for both correlation and bias, summarizing overall agreement in a single index; and Bland-Altman analysis for bias and limits of agreement (LoA).

Reliability metrics included the Cronbach α for internal consistency, and intraclass correlation coefficients (ICCs) were calculated to quantify (1) repeatability across the 5 independent GPT-o1 Pro and GPT-5 Pro runs (stability of scores when the same model was applied repeatedly to the same transcript) and (2) interrater reliability across the 5 physician raters (agreement among different human raters). A 2-sided α of <.05 denoted significance.

### Ethical Considerations

Ethics approval was obtained from the Juntendo University institutional review board (approval E24-0314-U02). All participants provided written informed consent before taking part. To protect participants’ privacy and confidentiality, all interview transcripts and performance scores were deidentified prior to evaluation and analysis by assigning study IDs and removing any potentially identifying information. Only deidentified transcripts were shared with the physician raters, and results are reported in aggregate. Study data were stored on password-protected, access-restricted institutional systems, and only the research team had access. Participants received no financial compensation for participation.

## Results

### Participant Scores

Table 1 summarizes the interview scores obtained via ABA-o1, ABA-5, and HBA. Across all 7 participants, group-level means were 53.7 (SD 6.8) for HBA, 53.2 (SD 9.2) for ABA-5, and 52.1 (SD 6.9) for ABA-01. Within-participant variability (mean CV percentage) was similar for the 2 automated methods (ABA-o1=6.6%; ABA-5=6.6%) and higher for HBA (13.9%). Individual-level differences were generally small, although notable divergences arose for RP 2 when comparing HBA vs ABA-o1 (46.8 vs 53.4; Δ=6.6) and for AP 2 when comparing ABA-5 vs HBA (67.8 vs 58.8; Δ=9.0) and ABA-5 vs ABA-o1 (67.8 vs 55.6; Δ=12.2).

**Table 1.** Mean scores by method and participant (n=7).

Participant	HBA^a^		ABA-o1^b^		ABA-5^c^	
	Score (0-125), mean (SD)	CV^d^ (%)	Score (0-125), mean (SD)	CV (%)	Score (0-125), mean (SD)	CV (%)
Medical student 1	48.0 (8.9)	18.5	46.4 (2.4)	5.2	46.0 (1.9)	4.1
Medical student 2	65.0 (9.7)	15.0	63.6 (5.1)	8.1	64.6 (4.2)	6.5
Resident physician 1	47.0 (2.9)	6.2	46.8 (2.9)	6.1	50.0 (2.6)	5.3
Resident physician 2	53.4 (7.2)	13.4	46.8 (3.3)	7.2	51.0 (7.1)	14.0
Resident physician 3	47.2 (3.6)	7.6	47.6 (2.7)	5.7	44.0 (1.0)	2.3
Attending physician 1	56.4 (9.4)	16.7	58.0 (5.4)	9.3	49.2 (2.6)	5.3
Attending physician 2	58.8 (11.7)	19.8	55.6 (2.7)	4.9	67.8 (6.2)	9.1
All	53.7 (6.8)	13.9	52.1 (6.9)	6.6	53.2 (9.2)	6.6

a HBA: human-based assessment.

b ABA-o1: artificial intelligence–based assessment (ABA) using GPT-o1 Pro.

c ABA-5: ABA using GPT-5 Pro.

d CV: coefficient of variation.

### Agreement and Reliability Across ABA-o1, ABA-5, and HBA

Agreement and reliability were evaluated across the 3 rating methods (ABA-o1, ABA-5, and HBA). Pairwise concordance with HBA was high for both AI variants: ABA-o1 vs HBA showed a Pearson correlation coefficient (*r*) of 0.90 (95% CI 0.78‐0.96) and CCC of 0.88; ABA-5 vs HBA showed an *r* of 0.87 (95% CI 0.72‐0.94) and CCC of 0.86. Concordance between the 2 AI pipelines was the highest (ABA-o1 vs ABA-5: *r*=0.98, 95% CI 0.95‐0.99; CCC=0.98), indicating near interchangeability of the AI variants (Table 2). Internal consistency followed the same pattern: Cronbach α was 0.81, 0.86, and 0.80 for ABA-o1, ABA-5, and HBA, respectively.

**Table 2.** Correlation, concordance, and internal consistency between artificial intelligence–based assessment (ABA) and human-based assessment (HBA) scores. Higher values indicate stronger association or consistency.

Comparison	Number of items	Pearson *r* (95% CI)	Lin CCC^a^
ABA-o1^b^ vs HBA	25	0.90 (0.78‐0.96)	0.88
ABA-5^c^ vs HBA	25	0.87 (0.72‐0.94)	0.86
ABA-o1 vs ABA-5	25	0.98 (0.95‐0.99)	0.98

a CCC: concordance correlation coefficient.

b ABA-o1: ABA using GPT-o1 Pro.

c ABA-5: ABA using GPT-5 Pro.

**Figure 1.**
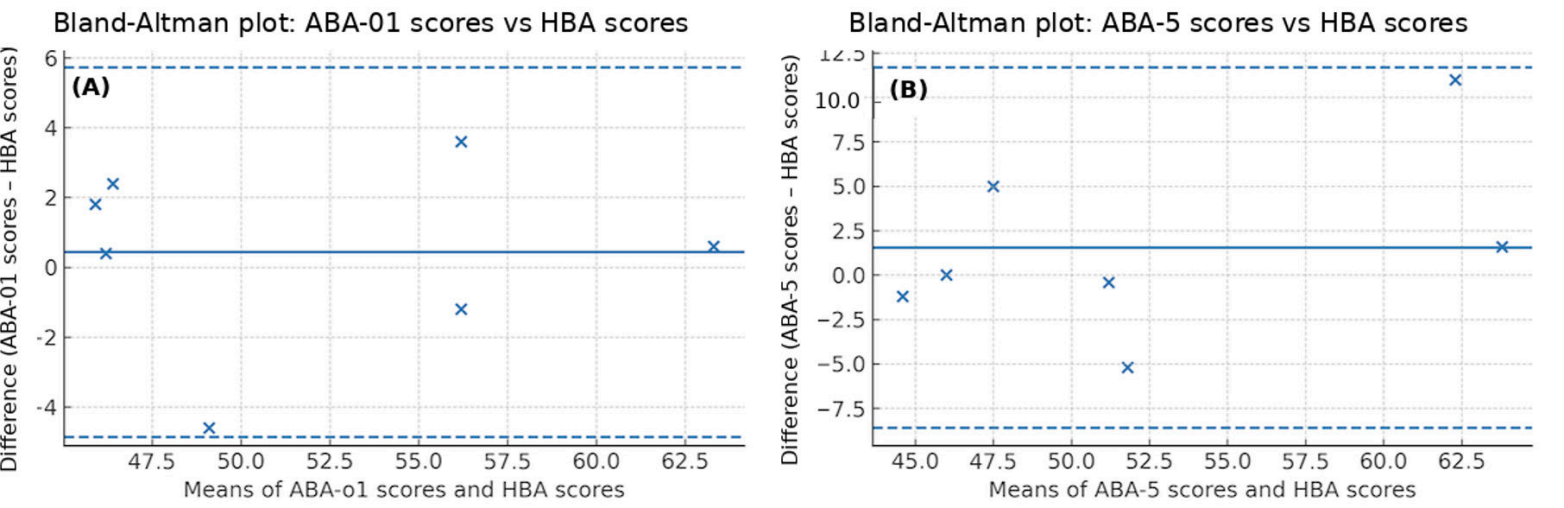
Bland-Altman plots comparing artificial intelligence–based assessment (ABA) with human-based assessment (HBA): (A) ABA using GPT-o1 Pro (ABA-o1) vs HBA (mean bias 0.43 [SD of differences 2.70]; limits of agreement [LoA]=−4.87 to 5.72) and (B) ABA using GPT-5 Pro (ABA-5) vs HBA (mean bias 1.54 [SD of differences 5.17]; LoA=−8.60 to 11.68). The points indicate participants (×). The solid line shows the mean bias; the dashed lines indicate the LoA.

All correlations were significant (2-sided *P*<.001). Bland-Altman analyses comparing each ABA with HBA showed small positive mean biases (ABA-o1 vs HBA: +0.43 [SD of differences 2.70]; ABA-5 vs HBA: +1.54[SD of differences 5.17]), with 95% LoA of −4.87 to 5.72 and −8.60 to 11.68, respectively; no proportional bias was observed in either comparison (Figures 1A and 1B).

Repeatability was assessed using the ICC. ABA-o1 showed substantial repeatability across 5 independent runs (ICC(3,1)=0.77; ICC(3,5)=0.94), and ABA-5 likewise showed substantial repeatability (ICC(3,1)=0.82; ICC(3,5)=0.96). In contrast, interrater reliability among the 5 HBA physician raters was only fair on single measures (ICC(2,1)=0.38) and improved when averaging them (ICC(2,5)=0.75). Overall, both AI-based approaches yielded more stable ratings across repeated evaluations than HBA, with ABA-5 slightly more stable than ABA-o1.

### Scores by Training Level

Table 3 summarizes mean interview scores and SDs by training level. Across methods, APs had the highest means (HBA: 57.6, SD 1.7; ABA-o1: 56.8, SD 1.7; ABA-5: 58.5, SD 13.2). MSs were next (HBA: 56.5, SD 12.0; ABA-o1: 55.0, SD 12.2; ABA-5: 55.3, SD 13.2), in some cases approximating AP performance. RPs had the lowest means (HBA: 49.2, SD 3.6; ABA-o1: 47.1, SD 0.5; ABA-5: 48.3, SD 3.8). Therefore, the anticipated ordinal pattern (APs>RPs>MSs) was not consistently observed as MS means exceeded RP means across all methods.

**Table 3.** Mean interview scores by training level as rated via human-based assessment (HBA) and artificial intelligence–based assessment (ABA).

Group	Participants per group, n	HBA score (0-125), mean (SD)	ABA-o1^a^ score (0-125), mean (SD)	ABA-5^b^ score (0-125), mean (SD)
Attending physicians	2	57.6 (1.7)	56.8 (1.7)	58.5 (13.2)
Medical students	2	56.5 (12.0)	55.0 (12.2)	55.3 (13.2)
Resident physicians	3	49.2 (3.6)	47.1 (0.5)	48.3 (3.8)

a ABA-o1: ABA using GPT-o1 Pro.

b ABA-5: ABA using GPT-5 Pro.

### Processing Time (35 Cases)

Total processing time was 5 hours, 59 minutes, 35 seconds for the physician benchmark; 1 hour, 56 minutes, 38 seconds for ABA-5; and 2 hours, 31 minutes, 5 seconds for ABA-o1. Average time per case was 3 minutes, 19.9 seconds for ABA-5 (batch-to-batch SD 1 minute, 6 seconds); 4 minutes, 19 seconds for ABA-o1 (SD 3 minutes, 9 seconds); and 10 minutes, 16.4 seconds for the physicians (SD 11 minutes, 9 seconds). Relative to the physicians, total time was reduced by 67.6% with ABA-5 and 58% with ABA-o1 (Table 4).

**Table 4.** Analysis time by method (5 independent runs and raters per method). “Batch-to-batch SD” indicates across-run variability. “Time reduction vs physicians” indicates the percentage reduction relative to human-based assessment (HBA).

Method	Total time	Mean time per case (batch-to-batch SD)	Time reduction vs physicians (%)
ABA-5^a^	1 h, 56 min, 38 s	3 min, 20 s (1 min, 6 s)	67.6
ABA-o1^b^	2 h, 31 min, 5 s	4 min, 19 s (3 min, 9 s)	58.0
HBA	5 h, 59 min, 35 s	10 min, 16 s (11 min, 9 s)	—^c^

a ABA-5: artificial intelligence–based assessment (ABA) using GPT-5 Pro.

b ABA-o1: ABA using GPT-o1 Pro.

c Not applicable.

## Discussion

### Principal Findings

In this validation study comparing 3 rater groups (HBA, ABA-o1, and ABA-5), ABA-o1 and ABA-5 produced interview ratings that were statistically indistinguishable from those produced via HBA yet showed markedly superior psychometric stability relative to HBA (Cronbach α: ABA-o1=0.81, ABA-5=0.86, and HBA=0.80; ICC: ABA-o1=0.77, ABA-5=0.82, and HBA=0.38). Cronbach α values of ≥0.8 indicate good internal consistency [10], and ICC(2,1) values of ≥0.75 denote good interrater reliability [11]. Agreement metrics were likewise robust as evaluative tools: the CCC assesses both correlation and bias in a single index [12], whereas Bland-Altman analysis remains the standard for visualizing bias and LoA [13]. ABA-5 was benchmarked against HBA using the same agreement framework.

Although the observed differences in reliability were significant, they may also have practical implications in educational settings. The consistently higher internal consistency and interrater reliability suggest that ABA scoring (including ABA-o1 and ABA-5) could enhance assessment efficiency and reproducibility. Depending on the context, ABA may serve not only as a scalable adjunct but also as a viable alternative to human raters in transcript-based clinical interview evaluations, although this requires significant larger-scale validation.

### Comparison With Prior Work

These findings corroborate previous work in which LLMs matched or exceeded faculty performance when scoring free-text notes [6], designing script concordance tests [7], and evaluating OSCE encounters [8]. A recent study showed that GPT-4o can produce inpatient documentation of comparable quality to that produced by resident physicians while reducing charting time by >50% [14]. In particular, studies of OSCE history-taking and free-text documentation have begun to demonstrate that LLM-based raters can apply communication-focused rubrics in virtual or simulated encounters with performance comparable to that of trained faculty, underscoring the relevance of AI-supported assessment in simulation-based learning contexts [2,6,8]. Consistent with ChatGPT’s passing performance on the US Medical Licensing Examination [15], this study suggests that foundation models possess clinically relevant semantic competence even in spoken communication tasks. Moreover, the 58% reduction in analysis time mirrors the 2025 Time for Class survey, where 36% of faculty who used generative AI daily reported a measurable workload decrease [16].

Beyond efficiency, such time savings could play a decisive role in addressing the growing problem of clinician educator burnout and faculty shortages, which are societal challenges that threaten the sustainability of CBME [17,18]. These concerns mirror prior reports that CBME assessment documentation requires several minutes per form and that the cumulative “assessment burden” is perceived as a threat to program sustainability [3,4]. By automating labor-intensive scoring, AI can free physicians to devote more time to high-value coaching and mentorship, thereby enhancing both educator well-being and learner support [17]. Furthermore, the superior scoring consistency observed with LLMs may help curb rater drift and cognitive biases such as leniency, halo, or contrast effects, which multicenter OSCE analyses have identified as long-recognized sources of unreliability and examiner-related variance in workplace-based assessments [19]. Improved fairness and reliability in assessment would advance equity in trainee progression and, ultimately, foster a more competent, patient-centered workforce.

### Interpretation and Educational Implications

From an educational perspective, 3 observations are noteworthy when framed across the 3 rater groups (HBA, ABA-o1, and ABA-5).

#### Consistency vs Nuance

The score distributions for ABA-o1 and ABA-5 suggest that these models apply the rubric more consistently than HBA raters, likely because their underlying embeddings execute the criteria more deterministically once sampling stochasticity is averaged across runs. Consistency is a hallmark of fair assessment; however, the absence of human nuance in ABA-o1 and ABA-5 could miss contextual subtleties (eg, cultural cues and atypical communication styles) that HBA raters may detect. Such subtleties may include culturally patterned ways of showing respect or disagreement, indirect or high-context communication, and unconventional but effective rapport-building strategies that are difficult to fully capture in a text-based rubric. Accordingly, this balance between reproducibility and subtlety is central when integrating ABA into educational programs; in our view, ABA is best used to enhance reproducibility and efficiency, with human raters remaining essential for high-stakes decisions and for cases in which subtle contextual factors are educationally or ethically salient.

#### Efficiency Gains

Relative to HBA (10 minutes, 16 seconds per case), ABA-5 and ABA-o1 reduced analytic time to 3 minutes, 20 seconds (–67.6%) and 4 minutes, 19 seconds (–58%) per case, respectively, amounting to approximately 240 and 210 faculty minutes saved across 35 encounters, respectively. In throughput terms, this corresponds to an increase in throughput from approximately 6 cases per hour with HBA to 18 cases per hour with ABA-5 and 14 cases per hour with ABA-o1, supporting more timely formative feedback and enabling the reinvestment of AI-derived efficiency gains into coaching rather than grading. When viewed alongside the lower CV and higher reliability indexes for AI-based scoring, these efficiency gains suggest that ABA could support more consistent and sustainable assessment practices within CBME frameworks [3,4,19]. In addition, ABA-5 could process data for 3 to 4 individuals in a single run, reducing the need for repeated prompt inputs and minimizing data handling overhead.

#### Level-Based Performance

MSs outperformed RPs on the same rubric in this cohort. This pattern may reflect (1) sampling error in a modest cohort, (2) case specificity favoring recently studied content, and/or (3) a rubric that emphasizes foundational communication more than advanced clinical reasoning. In particular, the MIRS prioritizes patient-centered communication behaviors that are heavily emphasized in undergraduate curricula and may be less sensitive to more advanced diagnostic reasoning skills typically developed during residency. Given the small number of participants and the single standardized case focused on a classic thyrotoxic periodic paralysis presentation, this unexpected pattern should be interpreted as a context-bound, hypothesis-generating finding rather than evidence that MSs generally outperform RPs in broader clinical performance. Replication with larger, more varied case sets and tiered rubrics evaluated across HBA, ABA-o1, and ABA-5 is warranted.

#### Practical Implications

Practically, programs could deploy an “AI-first, faculty-verified” workflow in which ABA-o1 and ABA-5 provide rapid formative scores and narrative feedback immediately after an encounter and HBA then audits a random subset for quality assurance, similar to double reading in radiology. Such hybridity leverages the speed and reliability of LLMs while retaining human oversight for high-stakes decisions.

### Strengths and Limitations

A key strength is the dual evaluation of accuracy (agreement) and efficiency (time), providing a more complete picture of implementation value than through accuracy alone. Nonetheless, several limitations warrant caution:

First, only 7 participants and a single thyrotoxic periodic paralysis scenario were tested, limiting generalizability across learner levels, languages, and clinical contexts. The small and homogeneous sample also restricts the psychometric interpretation of the findings; for example, differences in learner experience, case complexity, and language environment may influence both human and AI scoring behaviors. Therefore, these results should be viewed as preliminary and hypothesis generating rather than confirmatory. Second, convenience sampling and self-selection may have introduced bias toward technology-friendly participants. Third, model and prompt dependence was a limitation; the results correspond to GPT-o1 Pro and GPT-5 Pro with a specific rubric prompt; other LLMs or prompt engineering strategies could alter performance. Fourth, speech-to-text errors were not exhaustively audited and may have influenced ratings. In addition, the evaluation was limited to transcribed textual data; nonverbal cues, vocal tone, and conversational pauses present in the actual interviews could not be assessed. Fifth, there was potentially a systemic bias. High concordance does not preclude shared cognitive blind spots between AI and human raters; fairness audits across sex, accent, and cultural communication styles remain necessary. In practical implementations, this would entail periodic subgroup analyses of score distributions, qualitative review of discrepant cases, and predefined procedures for pausing or adjusting AI-based scoring if systematic disparities are detected.

### Future Research

Future studies should (1) evaluate multiple diverse clinical scenarios, including psychosocially complex cases; (2) compare real-time vs postencounter AI feedback; (3) examine learner outcomes such as skill acquisition and satisfaction; (4) conduct cost-effectiveness analyses at scale; and (5) develop and evaluate bias mitigation and explainability techniques—such as routine fairness dashboards, scheduled revalidation against human ratings, and faculty-led oversight processes—to satisfy accreditation requirements.

As this study was limited to transcript-based assessments of simulated encounters, future work is also needed to evaluate how well ABA scores correlate with actual clinical performance and whether AI can reduce assessor burden while maintaining fairness and reliability.

### Conclusions

Within the constraints of this pilot, GPT-o1 Pro and GPT-5 Pro matched expert physicians in scoring simulated patient interviews; produced more reliable ratings; and delivered a substantial 58% and 67.6% reduction in analytical time, respectively. These preliminary results indicate that LLMs could serve as a complementary or alternative tool to human raters for transcript-based interview assessments. This approach warrants further investigation as a means to contribute to assessment efficiency in medical education. Careful curricular design and continuous human oversight will be essential to ensure that such tools enhance rather than compromise the validity and equity of learner evaluations.

## Supplementary material

10.2196/81673 Multimedia Appendix 1 Prompt used for GPT-o1 Pro and GPT-5 Pro scoring of medical interview transcripts.

## Abbreviations

ABA: artificial intelligence–based assessment
ABA-5: artificial intelligence–based assessment using GPT-5 Pro
ABA-o1: artificial intelligence–based assessment using GPT-o1 Pro
AI: artificial intelligence
AP: attending physician
CBME: competency-based medical education
CCC: concordance correlation coefficient
CV: coefficient of variation
HBA: human-based assessment
ICC: intraclass correlation coefficient
LLM: large language model
LoA: limits of agreement
MIRS: Master Interview Rating Scale
MS: medical student
OSCE: objective structured clinical examination
RP: resident physician
